# Prediction of *Toxoplasma gondii* virulence factor ROP18 competitive inhibitors by virtual screening

**DOI:** 10.1186/s13071-019-3341-y

**Published:** 2019-03-13

**Authors:** Kun Yin, Guihua Zhao, Chao Xu, Xiao Qiu, Biwei Wen, Hui Sun, Gongzhen Liu, Ye Liu, Qingsong Zhao, Qingkuan Wei, Bingcheng Huang, Ge Yan, Jianping Cao

**Affiliations:** 1 Shandong Academy of Medical Sciences, Shandong Institute of Parasitical Disease, Jining, 272033 China; 2 Key Laboratory of Parasite and Vector Biology, Ministry of Health, Shanghai, 200025 China; 3 National Institute of Parasitic Diseases, Chinese Center for Disease Control and Prevention, WHO Collaborating Center for Tropical Diseases, Shanghai, 200025 China; 4 Jining No.1 People’s Hospital, Jining, 272000 China; 5 Department of Obstetrics and Gynecology, Shanghai Changhai Hospital, Second Military Medical University, Shanghai, 200433 China

**Keywords:** Virtual screening, Pharmacophore model, Competitive inhibitors, ROP18, *Toxoplasma gondii*

## Abstract

**Background:**

Rhoptry protein 18 (ROP18) is a key virulence factor of *Toxoplasma gondii*. The host’s immune responses mediated by immune-related GTPases (IRGs) could be blocked by ROP18’s kinase activity. ROP18 also interacts with various substrates, such as activating transcription factor 6 beta (ATF6β) and affects multiple physiological functions within host cells, thereby inducing intense virulence. In this study, competitive inhibitors targeted to ROP18 were subjected to virtual screening based on the principle of structure-based drug design (SBDD).

**Methods:**

The preparation of the ROP18 structure was conducted using the “Structure Prepare” function of Molecular Operating Environment (MOE) software. The ATP-binding pocket was selected as the starting point for virtual screening. Construction of the pharmacophore model used Extended Hückel Theory (EHT) half-quantitative measurement and construction, as well as the characteristics of Type I kinase inhibitors. The pharmacophore model of ROP18 was imported into the Specs database for small molecule similarity screening using EHT pharmacophore measurement. Hit compounds were selected using the functions of London dG and generalized-born volume integral/weighted surface area (GBVI/WSA) scoring. The top 100 hits were analyzed by molecular docking and structure activity relationships (SAR) analysis.

**Results:**

The final pharmacophore comprised three typical characteristics: three hydrogen bond acceptors/donors, two ring aromatic features occupying the hydrophobic core, and one cation group feature targeted to the terminus of ATP. A total of 1314 hit compounds analogous to ROP18 pharmacophore were passed through the Specs. After two rounds of docking, 25 out of 100 hits were identified as belonging to two main scaffold types: phthalimide ring structure, thiazole ring and styrene structure. Additionally, the screen also identified 13 inhibitors with distinct scaffold types. The docking models and SAR analysis demonstrated that these hits could engage in multiple hydrogen bonds, salt bridges halogen bonds, and hydrophobic interactions with ROP18, and para-position halo substituents on the benzene ring may enhance their affinity scoring.

**Conclusions:**

A pharmacophore against the ROP18 ATP-binding pocket was successfully constructed, and 25 representative inhibitors from 15 scaffold clusters were screened using the Specs database. Our results provide useful scaffold types for the chemical inhibition of ROP18 or alternative conformations to develop new anti-toxoplasmosis drug leads.

**Electronic supplementary material:**

The online version of this article (10.1186/s13071-019-3341-y) contains supplementary material, which is available to authorized users.

## Background

*Toxoplasma gondii* is an opportunistic pathogenic protozoan that obligately infects a wide range of warm-blooded hosts. According to epidemiological statistics, the infection rate of *T. gondii* in the world’s population is up to 30% [1]. The latest serological investigation results showed that the average infection rate of *T. gondii* in the Chinese population was 12.3% [2]. Although the majority of *Toxoplasma* infections in humans are asymptomatic, infection in most hosts causes a lifelong chronic infection and cysts are preserved in host’s brain, skeletal muscle, heart and other vital organs [3]. In addition, *T. gondii* can cause severe infections and complications such as retinitis retinae, encephalitis, and even death in hosts with immunodeficiency [4]. Notably, patients with a history of recessive *Toxoplasma* infection can be reinfected [3]. Therefore, the prevention, diagnosis and treatment of toxoplasmosis must be resolved worldwide.

A variety of *T. gondii* isolates is distributed worldwide with distinct virulences. The rhoptry of *T. gondii* is a specialized secretory organelle that secretes a set of rhoptry kinases and pseudokinases, which form the rhoptry protein 2 (ROP2) family. Representative members of the ROP2 family, such as ROP18, ROP5 and ROP17 have been identified as key factors of strains distributed in Europe and North America, and are associated with acute virulence [5–7]. In addition, ROP18 could play key roles in the virulence determination of a type I strain (T.gHB1) isolated from central China [8]. ROP18 is an active kinase that phosphorylates immunity-related GTPases (IRGs) of rodent hosts, such as Irga6, Irgb6 and Irgb10, which are upregulated by interferon-γ (IFN-γ) and act as the main mechanism for clearance of susceptible strains with moderate virulence [9–11]. ROP18 also phosphorylates a host endoplasmic reticulum bound transcription factor, activating transcription factor 6 beta (ATF6β) [12, 13] and a human p65 guanylate binding protein 1 (GBP1) factor [14], thus maintaining the integrity of the parasitophorous vacuolar membrane (PVM), and promoting the acute virulence of the corresponding isolates. Studies on factors that interact with ROP18 in host cells also indicated that ROP18 is associated with host cell apoptosis [15], protein degradation [16], reinfection of *T. gondii* and brain infections [3]. Therefore, ROP18 is a key participant in controlling virulence in both rodent and human hosts.

Given the importance of ROP18 in virulence determination, the present study aimed to screen competitive chemical inhibitors to block the kinase activity of ROP18 and prevent the acute virulence of type I strains. We performed a virtual screening study based on the crystal structure of ROP18. A conservative pharmacophore model was designed to target the ATP-binding pocket of the ROP18 kinase domain (KD). Ultimately, 25 hit compounds were identified from the Specs database. Structure–activity relationship (SAR) analysis of the 25 hits showed that the ROP18 inhibitors belong to two main chemical scaffolds and another 13 distinct scaffolds, with high virtual affinity scores (S score). The docking models of the hit compounds to ROP18 also revealed hot binding sites within the pocket. Our study provides scaffold types for ROP18 chemical inhibitors and thus lays a foundation to develop anti-toxoplasmosis drug leads.

## Methods

### Structure, software and databases

The three-dimensional (3D) structure of ROP18 was downloaded from the Research Collaboratory for Structural Bioinformatics (RCSB) Protein Data Bank (PDB) database (http://www.rcsb.org/pdb/home/home.do); the PDB code was 4JRN. MOE (version 2016.08; https://www.chemcomp.com/MOE2016.htm) software was used to preprocess the downloaded structure and perform the virtual screening. All chemical compounds were derived from the Specs screening database, which contains 202,919 compounds available for virtual screening (http://www.SPECs.net/). All pictures were created with MOE and PyMOL software (https://pymol.org/2/). A detailed introduction to MOE can be found at https://www.chemcomp.com.

### Structure conversion and preprocessing

4JRN was imported into MOE with the following parameters: the force field was Amber 10: EHT and the solvent model was R-Field. Correction of the structure and designation errors, repair of chain scission, protonation, and charge addition were conducted by the “Structure Prepare” module to prepare the structure. Optimization of the hydrogen bond network was accomplished using the Protonate 3D module. The prepared ROP18 complex structure was used in the subsequent steps.

### Active site selection

The sucrose-binding pocket and the ATP-binding pocket of 4JRN were analyzed using MOE and PyMOL software, respectively. The starting site for virtual screening was determined by a comparison of the volumes of the two pockets, amino acid properties, position, solvent accessible areas and hydrophobic/hydrophilic characteristics.

### Construction of the ROP18 pharmacophore model

The pharmacophore model against ROP18 was created on the basis of thorough interaction analysis of the residues that were around the β,γ-imidoadenosine 5′-triphosphate lithium salt hydrate (AMP-PNP) within 7 Å of the ATP-binding pocket. The conserved metals and water were retained in the binding pocket. The partial matching and exclude volumes were established in MOE for the subsequent steps of pharmacophore filtering. The pharmacophore model was created according to the EHT pharmacophore annotation and construction methods, and the intensity information of the ROP18 pharmacophore characteristics was determined using EHT semi-quantification. The features of the pharmacophore referred to the pharmacophore characteristics of typical Type I kinase inhibitors [17].

### Pharmacophore filtering (virtual screening)

A set of 202,919 compounds from the Specs database was used for virtual screening. In the first step, two-dimensional (2D) ligand-based searching using the Lipinski rules was performed. Subsequently, all the selected 2D compounds were transformed into a 3D conformation using Conformational Import. The remaining compounds (about 159,000) were imported into MOE using Pharmacophore Search and were screened against the ROP18 pharmacophore model according to the EHT notation. The compound conformations that were consistent with the pharmacophore features were reserved.

### Molecular docking

The crystal structure of native ROP18 (4JRN) was used for molecular docking to increase the efficiency and accuracy of virtual screening. The preliminary 5000 filtered compounds were initially docked to the ATP-binding pocket to eliminate false positive results. The docking force field was Amber10:EHT, compounds were placed using Triangle Matcher, and London dG (which estimates the free energy of ligand binding) was used as the first scoring function. Thereafter, the top 100 compounds were selected according with to their docking scores and conformations. After optimization of the force field, the top five conformations of the compounds that matched the pharmacophore model were retained. Then, GBVI/WSA (generalized-born volume integral/weighted surface area) was used as the second scoring function, the best conformation was saved as the output result, and the hit compounds were ranked by scoring functions, including cluster diversity and visual inspection. The docking models of compounds 1, 3 and 11 were also generated to display their hydrophobic, electrostatic, and hydrogen-bonding interaction potentials with ROP18. All docking models were generated using MOE and PyMOL software.

### SAR analysis

At the first step, the top 100 hit compounds according to the docking output results were analyzed manually. Then, SAR analysis of those compounds was performed using the function of SARreport. Analysis of the core scaffold and R-group was conducted and the S scores of the hits were calculated. Similarities between the interaction manner and core scaffolds were calculated using protein–ligand interaction fingerprint (PLIF) in MOE.

### Protein expression and purification

The recombinant ROP18 (187–554 aa) kinase domain was expressed in *Escherichia coli* Rosetta 2 (Novagen, Darmstadt, Germany) using the recombinant plasmid pETDL4.0-ROP18. The native ROP18 (187–554 aa) was expressed as a His6-GST-MBP-Gb1 fusion protein (GST: glutathione S-transferase, MBP: maltose-binding protein, Gb1: *Streptococcus* protein B1 domain) and batch purified on amylose beads, as previously described [18]. The purified protein was used directly for *in vitro* kinase assays.

### *In vitro* kinase reactions

Hit compounds 1, 3 and 11 were purchased from the Specs database. The solid biotinylated ROP18 substrate, as described previously [19], was synthesized by GL Biochem Shanghai Ltd. (Shanghai, China). The *in vitro* kinase activity of native ROP18 motif was detected using an HTRF KinEASE™ Kinase Kit (CISBIO, Codolet, France). The reaction buffer consisted of 5 mM MgCl_2_, 1 mM DTT, 1× enzymatic buffer, and 100 μM ATP. Each reaction contained 0.5 mM peptide substrate (three universal substrates STK-S1, STK-S2, STK-S3 or the solid ROP18 substrate). The recombinant ROP18 (187–554 aa) protein (1 ng/μl) was added to start the enzymatic step, and was incubated together with 1 mM hit compound for 30 min. The kinase reactions were incubated at room temperature for 50 min, then the prepared 1mAb-Eu(K) and 0.5 μM SA-XL665 mixture (10 μl/well) was added to stop the reaction. The products were detected on a Spectramax M5e Microplate Reader (Molecular Devices, Sunnyvale, America).

## Results

### Starting pocket selection for virtual screening

4JRN is a crystal structure of the ROP18 (187–554aa) KD containing two ligand-binding pockets. The sucrose-binding pocket of ROP18 is between the N-terminal 223–347 amino acids and is termed the ‘basic pocket’ [18]. The surface potentials in the core and termini of this region were all hydrophilic (Additional file 1: Figure S1a), corresponding with the two polar Arginines located in the termini of this region. Although this region has been identified as participating in the virulence determination of *T. gondii*, sucrose is not the natural substrate of ROP18 [18]. Moreover, analysis of the sucrose-ROP18 interface and surface potentials indicated that the four key interaction sites, Ser260, Gly261, Met284 and Arg347, form five pairs of hydrogen bonds with sucrose, and the surrounding amino acids are almost all polar residues, such as Arg223, Thr265, Ser285 and Glu286 (Additional file 1: Figure S1b). Therefore, the polarity of this basic pocket causes it to be directly exposed to the solution, and the hit compounds may be easily dissociated from the pocket.

**Fig. 1:**
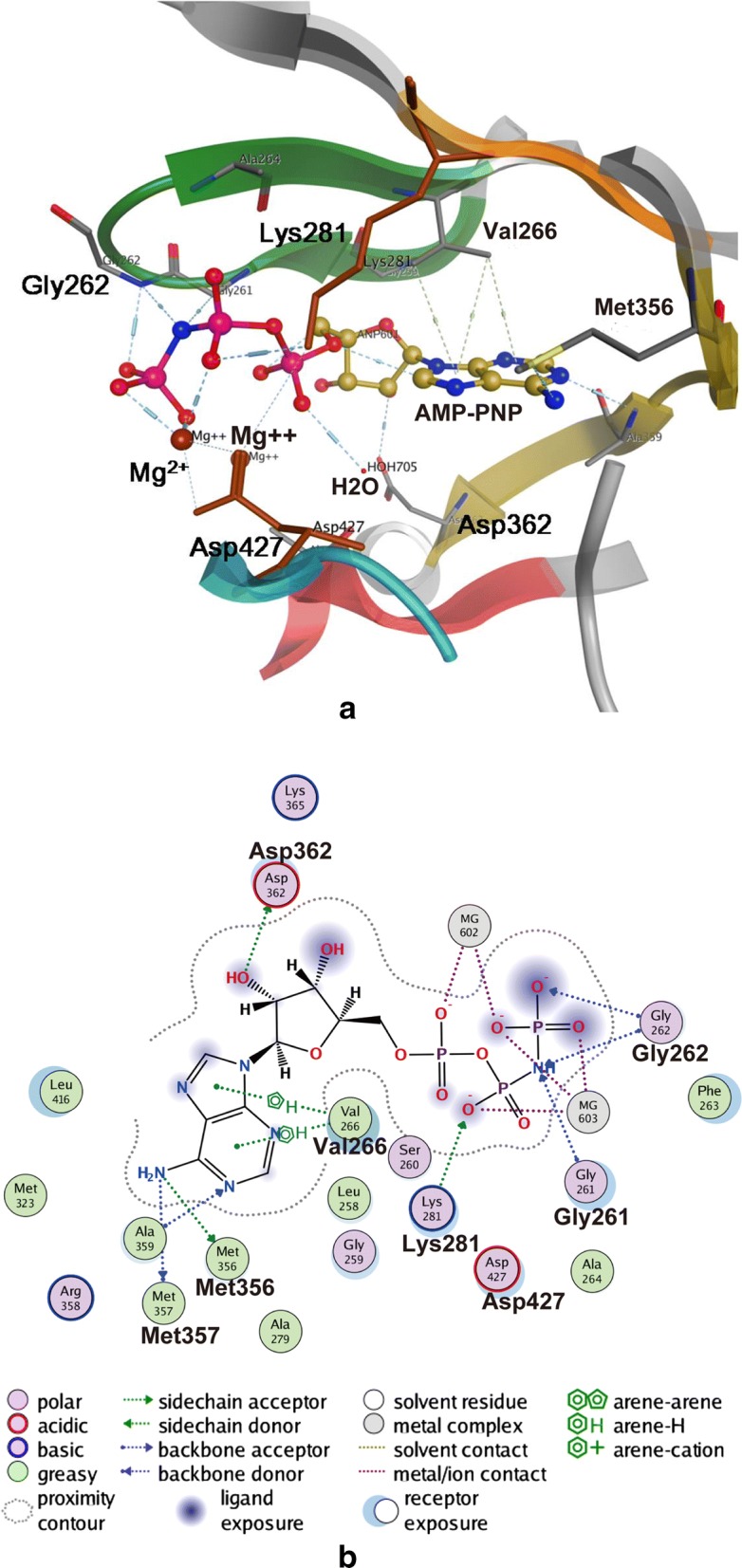
Interactions between ROP18 and AMP-PNP in the ATP-binding pocket. **a** Ribbon representation of the ROP18-AMP-PNP binding pocket. The hinge region is colored yellow, the DFG (Asp-Phe-Gly) is colored blue, the HRD (His-Arg-Asp) is colored red, the G-loop is colored green, the Hyd1 is colored orange, and the overall structure of ROP18 is colored grey. AMP-PNP is shown in a stick representation and colored spectrum rainbow. Residues interacting with AMP-PNP are shown in sticks. Hydrogen bonds are indicated as blue dashed lines and π-H interactions are indicated as green dashed lines. **b** Interaction schematic diagram of ATP-binding pocket in two-dimensional (2D) representation. Polar interactions are colored pinkish purple, greasy interactions are colored green, Mg^2+^ are colored grey, ligand exposures are colored violet, interactions between residues/Mg^2+^ and AMP-PNP are indicated as dashed lines. Hydrogen bonds are indicated as blue dashed lines, the π-H interactions are indicated as green dashed lines, and the metal/ion contacts are indicated as purple dashed lines. The directions of the arrows in dashed lines indicate hydrogen bond donors or side chain donors. *Abbreviations*: ROP18, rhoptry protein 18; AMP-PNP, β,γ-imidoadenosine 5′-triphosphate lithium salt hydrate

At the same time, the interaction pattern of ROP18-AMP-PNP (an analog of ATP) was consistent with the typical kinase-substrate interaction. In addition to ATP, this pocket also contains one molecule of H_2_O and two of Mg^2+^. The adenosine heterocycle of AMP-PNP forms a hydrogen bond with the O/N atom on the hinge region skeleton of ROP18, and the pentacyclic ring of ATP targets it to a hydrophobic pocket. The hydroxyl group forms a pair of hydrogen bonds with the negatively charged Asp362, and the negatively charged phosphate radical forms a pair of salt bridges and a coordinated bond with Mg^2+^. Meanwhile, Mg^2+^ forms a coordinated bond with Asp427, indicating that Mg^2+^ takes part in the interaction between the receptor and the ligand. The phosphate radical of ATP also forms a pair of hydrogen bonds with the N atom on the Gly262 skeleton (Fig. 1). The stable binding of Mg^2+^ and polar sites Asp362 and Lys281 ensures the specific binding of inhibitors. Based on the interaction analysis, we decided to anchor an inhibitor core in the ATP-binding pocket.

### The pharmacophore model against the ROP18 ATP-binding pocket

**Fig. 2:**
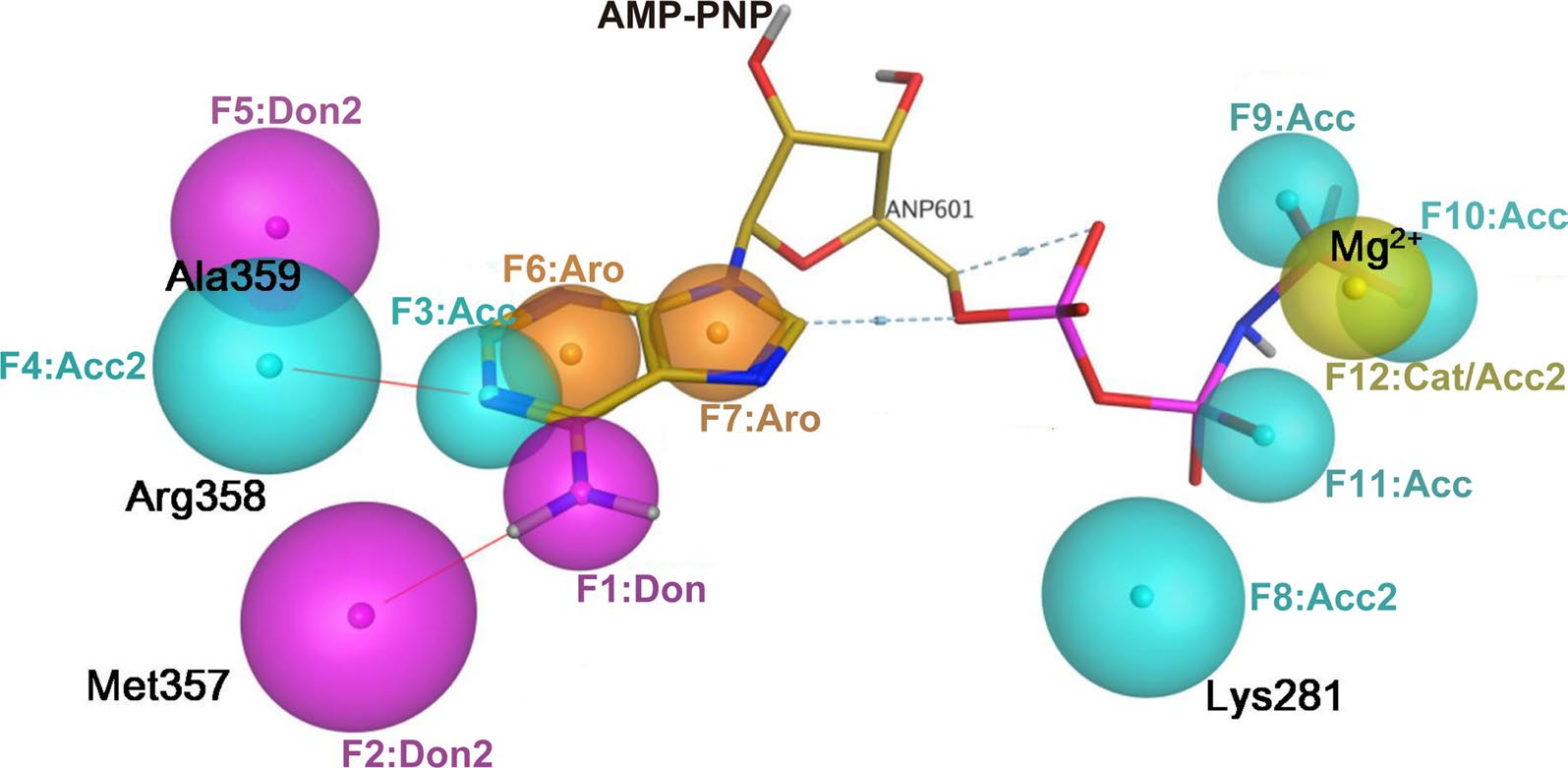
Pharmacophore model of ROP18 targeting to the ATP-binding pocket. The model contains a total of 12 features numbered F1–F12. AMP-PNP is shown in a stick representation and colored spectrum rainbow. Features of the pharmacophore model are shown in a semitransparent sphere. The hydrogen bond donor features are colored red, the hydrogen bond receptor features are colored blue, the ring aromatic features are colored orange and the cation group feature (Cat) is colored olive. *Key*: Acc: the hydrogen bond receptor of pharmacophore, Acc2: the hydrogen bond donor of residues in ROP18, Don: the hydrogen bond donor of pharmacophore, Don2: the hydrogen bond receptor of residues in ROP18. The exclude volume of this model is 1.7 Å. *Abbreviations*: ROP18, rhoptry protein 18; AMP-PNP, β,γ-imidoadenosine 5′-triphosphate lithium salt hydrate

A solid pharmacophore model of ROP18 was built in accordance with the characteristics of Type I inhibitors [17] and contains four pharmacodynamic features, numbered F1-F12 (Fig. 2). Based on the analysis results of a 3D description of the ATP-binding pocket (Additional file 2: Figure S2), there are more than four potential interaction sites within the hinge, such as Met357, Arg358, Ala359 and Ala279, which could serve as the hydrogen bond donors or acceptors for competitive inhibitors (Additional file 2: Figure S2a). Hence, the first type of feature is three potential hydrogen bonds at the backbone of the hinge region (Ala279, Met357, Arg358 and Ala359) and partial matching was set to satisfy at least one (F1-F5). The second type of feature has two hydrophobic features at the adenine ring of ANP, which serves as a core scaffold for side chains that occupy the adjacent hydrophobic regions (F6 and F7). Considering that Mg^2+^ participates in the interaction between the AMP-PNP phosphate radical and Asp427/Lys281 (Additional file 2: Figure S2b), which is likely to bind with Apo-ROP18, the third type of pharmacophore feature is three hydrogen donors or acceptors at the tail of AMP-PNP that mimic the hydrogen bonds and a cation group feature that function as a salt-bridge for phosphate (for Mg^2+^ and Lys281, F8-F12). The subjacent Mg^2+^ and the H_2_O molecule in complex with ROP18 were deleted before pharmacophore construction because of their non-compact interaction with the ligands (Additional file 2: Figure S2c, d). Finally, an excluded volume of 1.7 Å as a constraint in the receptor atoms was established to remove clashing ligands.

### Virtual screening and docking results in the Specs database

The ROP18 pharmacophore model was filtered in the Specs database, which contains 202,919 compounds. EHT scoring was used for pharmacophore scoring and about 15,782 compounds from the Specs databases were retained. After pharmacophore filtering, the top 5000 compounds were promoted into the next refined docking to rank their priority and to search for the rational binding models of the hit compounds. Classical triangle matching was chosen as the placement method and the force field was employed for pose refinement. We identified 1314 initial hit compounds when filtering using the pharmacophore model. The receptor-ligand affinity was evaluated using London dG and GBVI/WSA dG scores. After this step, the top 100 compounds were selected and docked into ROP18 manually. Finally, 25 out of the 100 hit compounds were identified as ROP18 competitive inhibitors according to the scoring rank. A flow chart showing the process of complete virtual screening applied in this study is provided in Additional file 3: Figure S3.

### SAR analysis of 25 hits

**Fig. 3:**
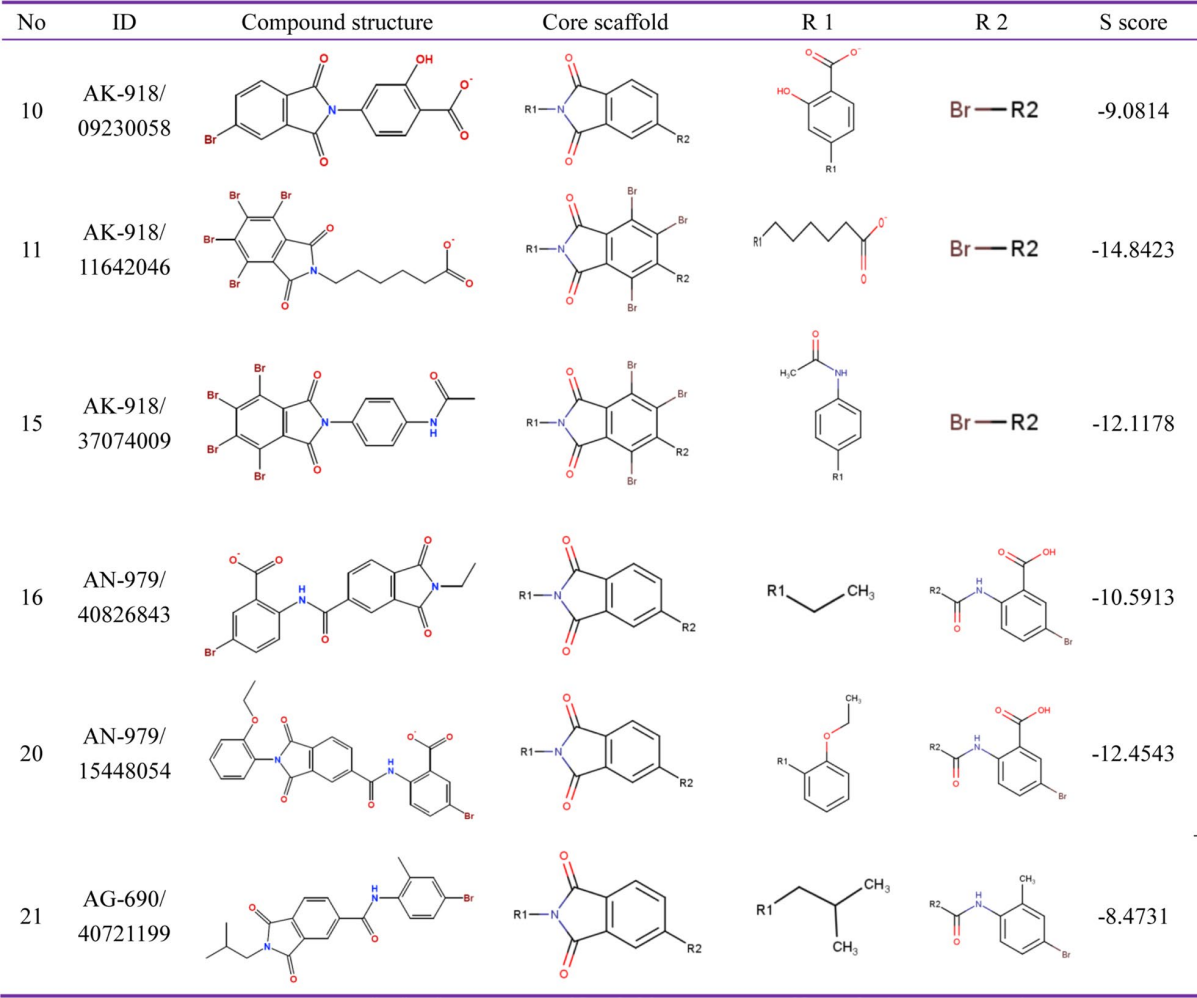
Summary of ROP18 inhibitors belonging to Scaffold type I. Their Specs accession number are shown as ID, R groups are shown as R1 and R2, S score represents the predicted affinity activities by MOE, and the higher negative values indicate the stronger affinity

**Fig. 4:**
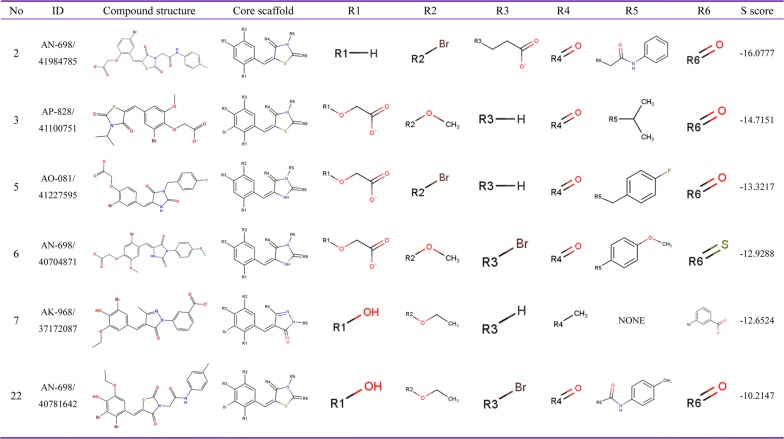
Summary of ROP18 inhibitors belonging to Scaffold type II. Their Specs accession number are shown as ID, R groups are shown as R1 to R6, S score represents the predicted affinity activities by MOE, and the higher negative values indicate the stronger affinity

**Fig. 5:**
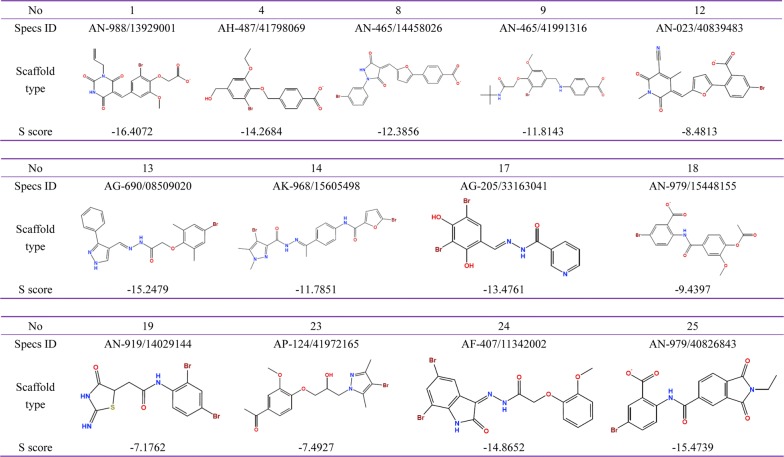
The scaffold types and S scores of the other 13 competitive inhibitors with distinct core scaffolds. Their Specs accession number are shown as the Specs ID, the core scaffold types are shown as chemical structure of those hit compounds. S score represents the predicted affinity activities by MOE, and the higher negative values indicate the stronger affinity

The representative scaffold type was calculated using MOE SAReport, and the core scaffold and R-group analysis were based on the calculation of PLIF. The final representative compounds were decided on the basis of comprehensive analysis, including the diversity types of the scaffolds, R-groups and S scores. The results identified two main representative scaffold types in view of their similar interaction manners and stem structures. Compounds 10, 11, 15, 16 and 20 have a similar phthalimide ring and were named as Scaffold type I (Fig. 3). Compounds 2, 3, 5, 6, 7 and 22 have a similar thiazole ring and styrene structure, and were named as Scaffold type II (Fig. 4). The affinity scores and R-groups of those hits are also displayed in Figs. 3 and 4. The remaining 13 hit compounds had distinct scaffold types (Fig. 5) and interaction styles.

**Fig. 6:**
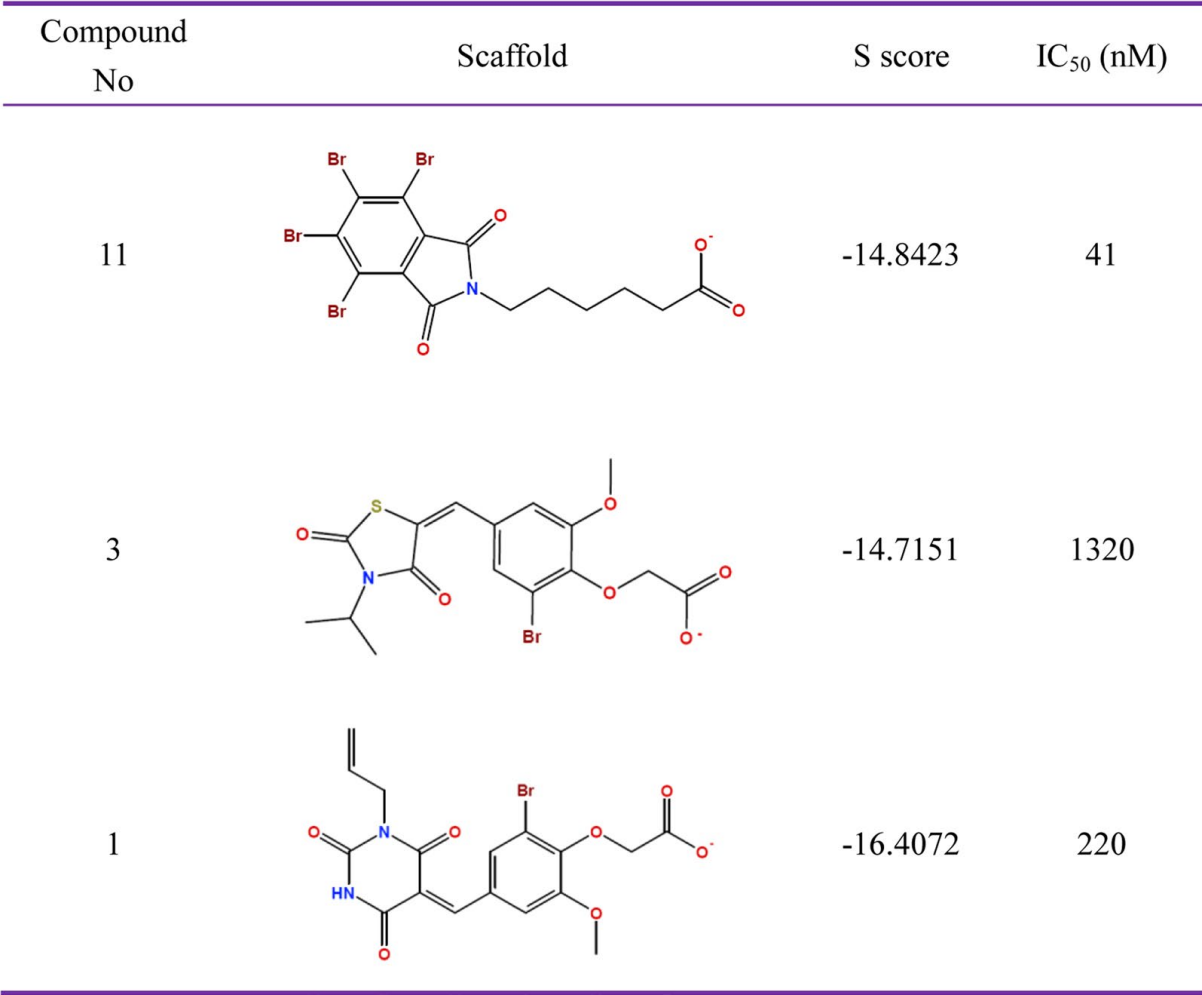
MOE predicted affinity value (S score) and experimentally detected IC_50_ of the three representative hit compounds (Compound 11, 3 and 1)

To validate the predictive affinity power of the hit compounds, the KD of ROP18 protein was purified and the three representative compounds (11, 3 and 1) were prepared for kinase reactions *in vitro*. Compound 11 showed the highest IC_50_ values of 41 nM. Compound 3 and compound 1, showed IC_50_ values of 1320 and 220 nM, respectively (Fig. 6).

### Docking model of compound 11 bound to ROP18

**Fig. 7:**
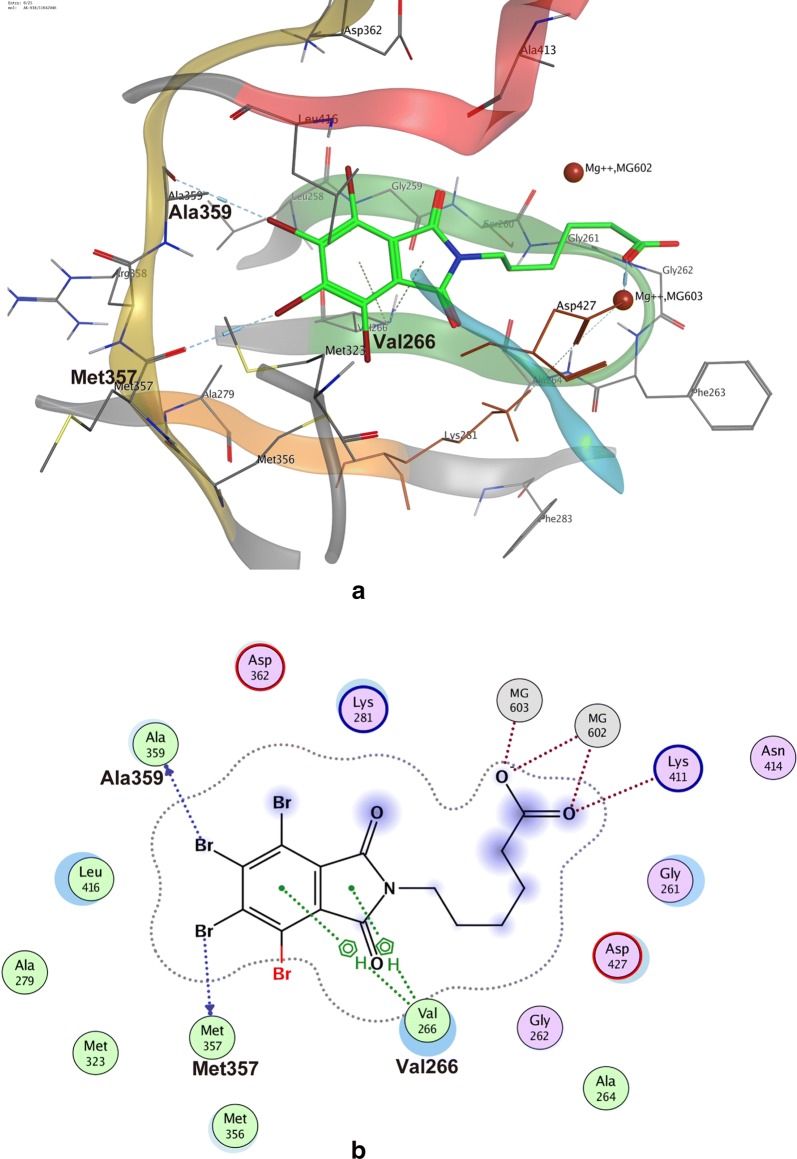
Docking model of compound AK-918/11642046 (No. 11) bound to ROP18. **a** Ribbon representation of the three-dimensional (3D) interface between compound 11 and ROP18. **b** Interaction schematic diagram of compound 11 in a two-dimensional (2D) representation. The coloring of the elements and the meaning of the dashed lines are the same as in Fig. 1. *Abbreviation*: ROP18, rhoptry protein 18

The molecular docking results indicated that the interaction styles and core scaffolds in Scaffold type I have highly similar characteristics. Compound 11 (ID: AK-918/11642046) had the highest S score. The docking model showed that compound 11 occupies the ATP binding pocket of ROP18, the hydrophobic ring and aromatic nucleus of the phthalimide scaffold anchors in the hydrophobic pocket, replacing the adenosine ring of ATP, and making a double H-π bond with the backbone of Val266. Hydrogen bonds are also engaged with the surrounding side-chains at the hinge region. The halo substituents at sites 5 and 6 on the phthalimide scaffold form two pairs of halogen bonds between the carbanyl groups at the backbones of Met357 and Ala359. Additionally, the carboxyl group in the tail of compound 11 forms a strong ionic bond with Mg^2+^, which would increase its binding affinity (Fig. 7). The other five compounds of this scaffold type, which share the phthalimide core, but have differences in their R-groups, showed different affinities. Loss of the para-position halo substituent on the benzene ring may explain the lower S scores of the other five compounds.

### Docking model of compound 3 bound to ROP18

**Fig. 8:**
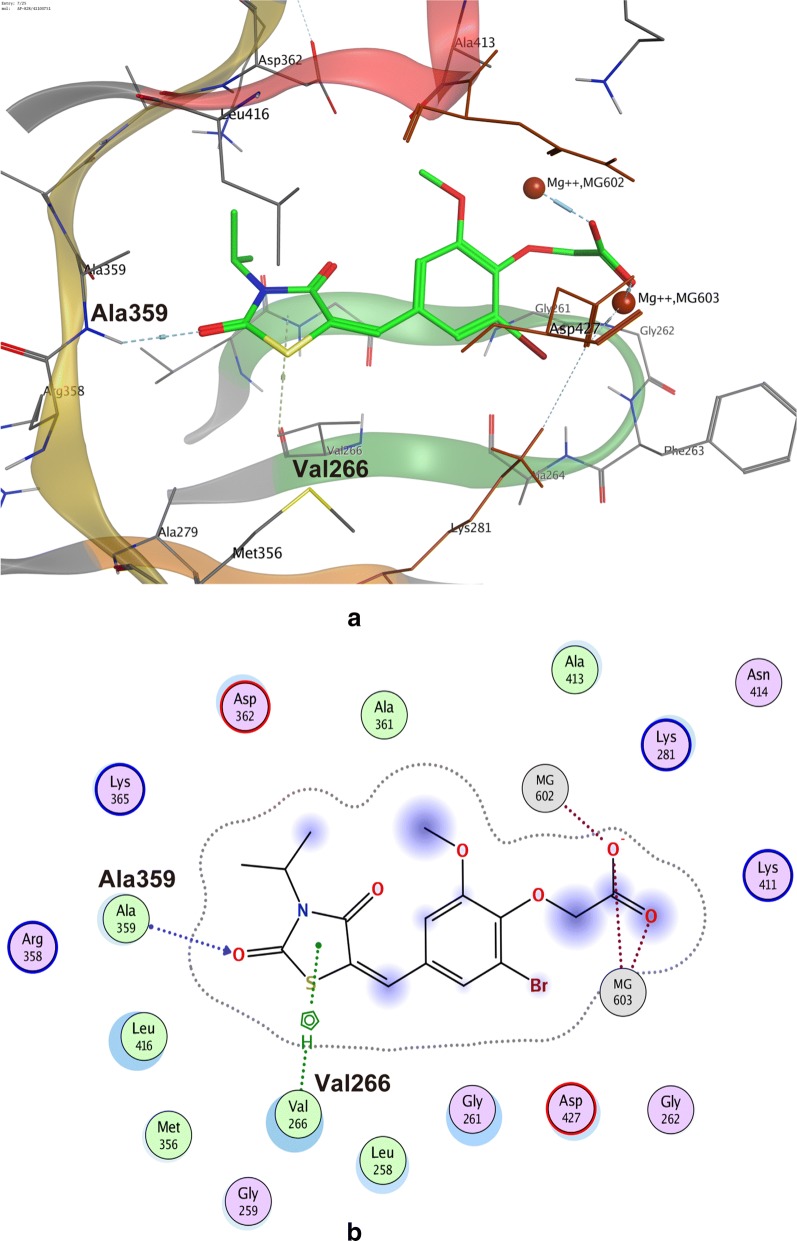
Docking model of compound AP-828/41100751 (No. 3) bound to ROP18. **a** Ribbon representation of the three-dimensional (3D) interface between compound 3 and Rop18. **b** Interaction schematic diagram of compound 3 in a two-dimensional (2D) representation. The coloring of the elements and the meaning of the dashed lines are the same as in Fig. 1. *Abbreviation*: ROP18, rhoptry protein 18

Compound 3 (ID: AP-828/41100751) had the highest S score among the Scaffold type II compounds and was selected to be docked to ROP18. The docking results indicated that the major thiazole ring occupies the ATP binding pocket of ROP18, replacing the adenosine ring of ATP. Compound 3 forms a pair of hydrogen bonds with the amino group of Ala359 at the hinge of ROP18, and also forms an H–π interaction with Val266. The carboxyl group at the other end of compound 3 forms two pairs of ionic bonds between the two Mg^2+^ ions, and also forms a pair of ionic bonds with the positively charged Lys411, further enhancing its affinity (Fig. 8). The R groups of this scaffold type are diverse and show different interactions, which might explain their different affinities.

## Discussion

*Toxoplasma gondii* is dominated by three clonal lineages (type I: acute virulence, type II: moderate virulence, and type III: weak virulence) [20]. Saeij et al. [5] and Taylor et al. [21] discovered that only the *rop18* loci showed obvious gene polymorphism (14%) among the three genotypes [21], whereas the differences among the other homologous genes were no more than 1% [22]. Therefore, ROP18 is one of the most important virulence determinants of *T. gondii*. El Hajj et al. [23] proved that amino acids 243–539 of ROP18 carry the serine/threonine protein kinase activity *in vitro*. Consistent with this, the crystal structure 4JRN adopts a typical folding pattern of a serine/threonine kinase. The functional domains, such as G-loop, hinge region, DFG (Asp-Phe-Gly) and HRD (His-Arg-Asp) all show stable folding. Recently, protein kinases have become popular drug targets to develop therapeutic agents to treat human diseases [24]. Most of the inhibitors target the ATP-binding pocket and are competitive inhibitors. Previous studies showed that ROP18 directly phosphorylates host factors and participates in the host immune response; hence, its serine/threonine KD is an ideal target to design anti-toxoplasmosis drugs. Additionally, the DFG region of ROP18 faces towards the ATP-binding pocket and thus the native ROP18 is in an ‘active’ state, indicating that this pocket is oriented correctly for inhibition by Type I inhibitors [17]. This type of inhibitor constitutes the majority of ATP-competitive inhibitors, such as sunitinib, which targets c-Src and Bcr-Abl kinases; and gefitinib and erlotinib, which target the epidermal growth factor receptor (EGFR) kinase. They can recognize the so-called active conformation of the kinase and typically consist of a heterocyclic ring system that presents one to three hydrogen bonds to the amino acids located at the hinge region [17]. The pharmacophore constructed in this study was in accordance with the characteristics of the type I inhibitors, and obtained a quantitative score by the semi-quantitative EHT, thus making it more accurate than the routine pharmacophore model.

In the present study, SAR analysis sorted the 25 hits into two major scaffold types and 13 distinct scaffold types. Based on the docking model and S score, the hits belonging to Scaffold type II show higher affinity activities than those of Scaffold type I. Compared with Scaffold type I, the core thiazole ring and styrene structure shared in Scaffold type II could form more hydrogen and ionic bonds, which may lead to compounds with improved affinity. However, the results of *in vitro* IC_50_ values of compound 11 and compound 3, combined with the R-group analysis, indicated that the para-position halo substituent on the benzene ring of compounds in Scaffold type I could enhance the interaction with ROP18 via halogen bonds, which have a stronger affinity than hydrogen bonds, thus reinforcing the interactions between the phthalimide ring scaffold and ROP18.

Structure similarity results of 4JRN were acquired with the RCSB PDB Comparison Tool [25]. Except for the structures of rhoptry proteins, such as ROP5, and a calcium dependent protein kinase 2 (CDPK2) expressed by *Plasmodium falciparum*, the alignment results showed that the structure of human checkpoint kinase 1 (Chk1) [26] (PDB ID: 2YEX) had the highest RMSD value of 2.75 compared with 4JRN, and the structure of human CaMKKβ (PDB ID: 2ZV2) [27] is also reminiscent of 4JRN (Additional file 4: Figure S4). An interaction comparison between 2YEX and 4JRN indicated an apparent difference in their ligand binding modes. In the complex of 2YEX, the Chk1 inhibitors triazoloquinolones/triazolones (TZs) are buried in a cluster of hydrophobic residues (Leu15, Val23, Gly90 and Leu137), and only form three hydrogen bonds with Glu85 and Cys87. The majority of the interacting residues are located outside of the structurally equivalent region (Additional file 4: Figure S4). Furthermore, Leu417_ROP18_ is not involved in the interaction with AMP-PNP, although it is structurally similar to Leu137_Chk1_. Similarly, the interactions between STO-609 and CaMKKβ are mostly hydrophobic, involving Ile172, Val180, Val250 and Phe268 from the N-lobe; and Gly274, Pro274, Leu320 and Asp331 from the C-lobe. In addition, there are only two STO-609 hydrogen bonds with the backbones of Val271 and Asp331 (Additional file 4: Figure S4). Kinase assay results proved that the CaMKKβ residue Pro274, which replaces the conserved acidic residue of other protein kinases, is an important determinant for selective inhibition by STO-609 [27]. Thus, the only identical residue Asp331_CaMKKβ_/Asp431_ROP18_ is not the key binding site. In addition to the distinct interaction mode, the majority features of the pharmacophore model are hydrogen bond interactions and the corresponding residues are different to those of Chk1 and CaMKKβ. Furthermore, the Mg^2+^-binding site and the volume limitation of the shape feature established in this model could ensure the specificity of hit compounds. Hence, the customized pharmacophore model of ROP18, the molecular similarity principle of screening, and the low homology to mammalian kinases would ensure the specificity of the hit compounds screened in this study.

Simpson et al. [19] identified 16 small molecule inhibitors that block the activity of ROP18 using a high-throughput screening assay. The substrate peptides, based on a native substrate of ROP18, were used for screening via microfluidic capillary electrophoresis (MCE). The hit compounds in that study belonged to three chemical scaffolds: oxindoles, 6-azaquinazolines and pyrazolopyridines. Compounds 1, 2, 7 and 11 in that study have similar core scaffolds to those discovered in the present study. In particular, compound 2 has the lowest IC_50_ value and can make multiple hydrogen bonds with residues Met357, Ala359, Asp362, Lys365 and Lys281 of ROP18 [27]. The core scaffold and the detailed interactions are similar to our hit compound 11. In the present study, sites 5 and 6 on the phthalimide ring were substituted by halogens and so formed two pairs of stronger halogen bonds between the backbone of Met357 and Ala359. This also indicated that our results are reliable; however, the inhibitory activity of our hits requires further investigation. In addition, the other 13 hit compounds in this study have different core scaffolds and interaction manners, indicating that our pharmacophore model is not only conservative, but also is flexible enough to ensure a diversity of hits. These diverse interactions increase the chances of discovering competitive inhibitors for ROP18.

## Conclusions

We constructed a pharmacophore model against the ATP-binding pocket of ROP18 and identified 25 small molecular inhibitors using virtual screening and SBDD. The hit compounds belong to two major scaffolds and 13 other distinct scaffolds. Docking modeling combined with SAR analysis demonstrated that the present hits could engage in complementary binding interactions comprising multiple hydrogen bonds, salt bridges, halogen bonds and hydrophobic interactions. These compounds provide useful scaffold types for ROP18 chemical inhibition or represent alternative lead conformations to develop therapeutics to treat toxoplasmosis.

## Additional files

**Additional file 1: Figure S1.** Interactions between ROP18 and sucrose in the sucrose-binding pocket. **a** Surface potential of the sucrose-binding pocket. The overall structure of ROP18 is shown in spheres with electrostatic surface potentials. Sucrose and AMP-PNP are shown as sticks. **b** Interaction schematic diagram of sucrose-binding pocket in a two-dimensional (2D) representation. The coloring of the elements and the meaning of the dashed lines are the same as in Fig. 1b. *Abbreviations*: ROP18, rhoptry protein 18; AMP-PNP, β,γ-imidoadenosine 5′-triphosphate lithium salt hydrate.

**Additional file 2: Figure S2.** Three-dimensional representation of the ROP18 ATP-binding pocket. Residues involved in the ligand-ROP18 interactions are shown as a sticks representation and the coloring of the kinase elements is the same as in Fig. 1a. AMP-PNP is shown in sticks and colored spectrum rainbow. Hydrogen bonds are indicated as blue dashed lines and π-H interactions are indicated as green dashed lines. **a** Sticks representation of the ROP18-AMP-PNP binding pocket. **b** Interaction details of the oben Mg^2+^. **c** Interaction details of the subjacent Mg^2+^. **d** Interaction details of the H_2_O molecule.

**Additional file 3: Figure S3.** Virtual screening protocol of the structure based approach for ROP18 pharmacophore model construction and competitive inhibitors identification.

**Additional file 4: Figure S4.** Interaction schematic diagrams of three structures of kinase-ligand complexes (4JRN, 2YEX and 2ZV2), the structure similarity and sequence alignment of the KDs of human kinases Chk1/CaMKKβ and ROP18, respectively. The black dashed lines in the interaction schematic diagrams indicate hydrogen bonds, salt bridges and metal interactions. Green solid lines show the hydrophobic interactions and green dashed lines show π-π and π-cation interactions. *Abbreviations*: KD, kinase domain; Chk2; checkpoint kinase 2; CaMKKβ, calcium/calmodulin dependent protein kinase kinase 2 beta; RMSD, root-mean-square deviation.

## Abbreviations

2D: two-dimensional
3D: three-dimensional
ATF6β: activating transcription factor 6 beta
CDPK2: calcium-dependent protein kinase 2
EGFR: epidermal growth factor receptor
EHT: extended Hückel theory
GBP1: guanylate binding protein 1
GBVI/WSA: generalized-born volume integral/weighted surface area
GST: glutathione S-transferase
IFN-γ: interferon-γ
IRGs: immune-related GTPases
KD: kinase domain
MBP: maltose-binding protein
MCE: microfluidic capillary electrophoresis
MOE: molecular operating environment
PDB: Protein Data Bank
PLIF: protein-ligand interaction fingerprint
PVM: parasitophorous vacuolar membrane
RCSB: Research Collaboratory for Structural Bioinformatics
ROP: rhoptry protein
SAR: structure activity relationships
SBDD: structure-based drug design
