# Supplementary figures and images for: Prediction of Toxoplasma gondii virulence factor ROP18 competitive inhibitors by virtual screening
Source: Parasit Vectors. 2019 Mar 13;12:98. doi: 10.1186/s13071-019-3341-y (PMC6416898; doi:10.1186/s13071-019-3341-y)

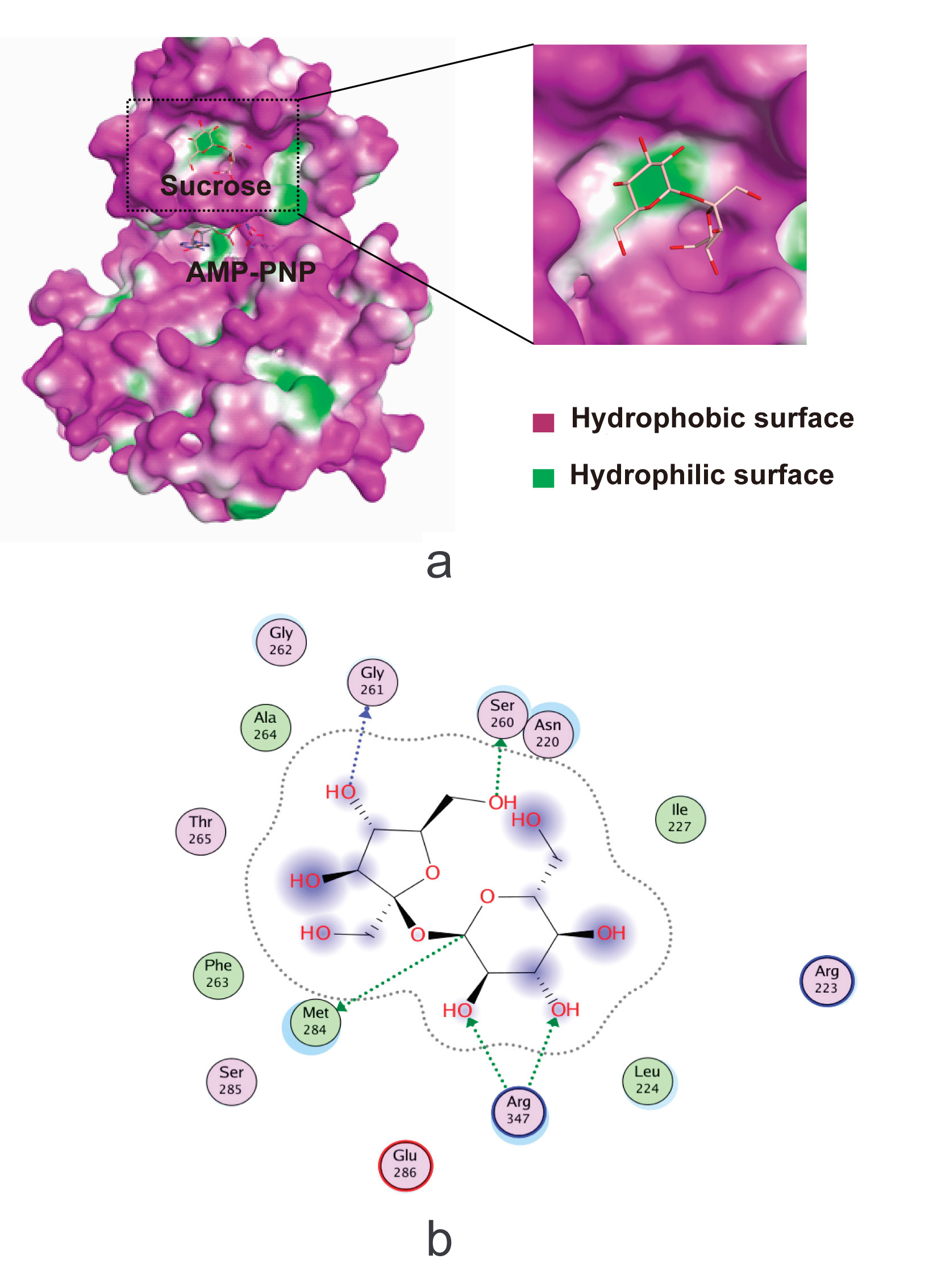

Supplement: Supplementary file 1 — Additional file 1: Figure S1. Interactions between ROP18 and sucrose in the sucrose-binding pocket. a Surface potential of the sucrose-binding pocket. The overall structure of ROP18 is shown in spheres with electrostatic surface potentials. Sucrose and AMP-PNP are shown as sticks. b Interaction schematic diagram of sucrose-binding pocket in a two-dimensional (2D) representation. The coloring of the elements and the meaning of the dashed lines are the same as in Fig. 1b. Abbreviations: ROP18, rhoptry protein 18; AMP-PNP, β,γ-imidoadenosine 5′-triphosphate lithium salt hydrate. [file 13071_2019_3341_MOESM1_ESM.tiff]

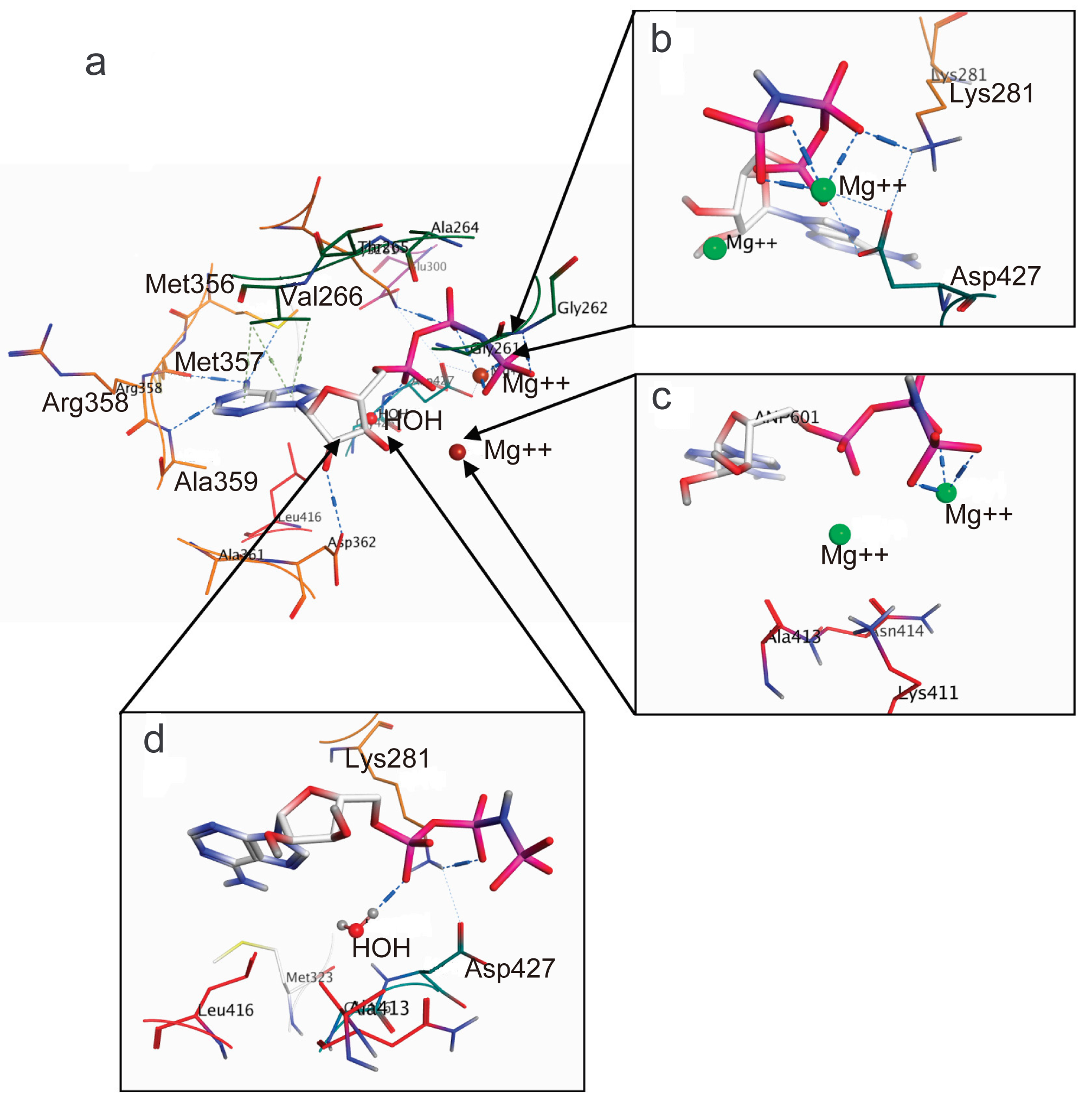

Supplement: Supplementary file 2 — Additional file 2: Figure S2. Three-dimensional representation of the ROP18 ATP-binding pocket. Residues involved in the ligand-ROP18 interactions are shown as a sticks representation and the coloring of the kinase elements is the same as in Fig. 1a. AMP-PNP is shown in sticks and colored spectrum rainbow. Hydrogen bonds are indicated as blue dashed lines and π-H interactions are indicated as green dashed lines. a Sticks representation of the ROP18-AMP-PNP binding pocket. b Interaction details of the oben Mg2+. c Interaction details of the subjacent Mg2+. d Interaction details of the H2O molecule. [file 13071_2019_3341_MOESM2_ESM.tiff]

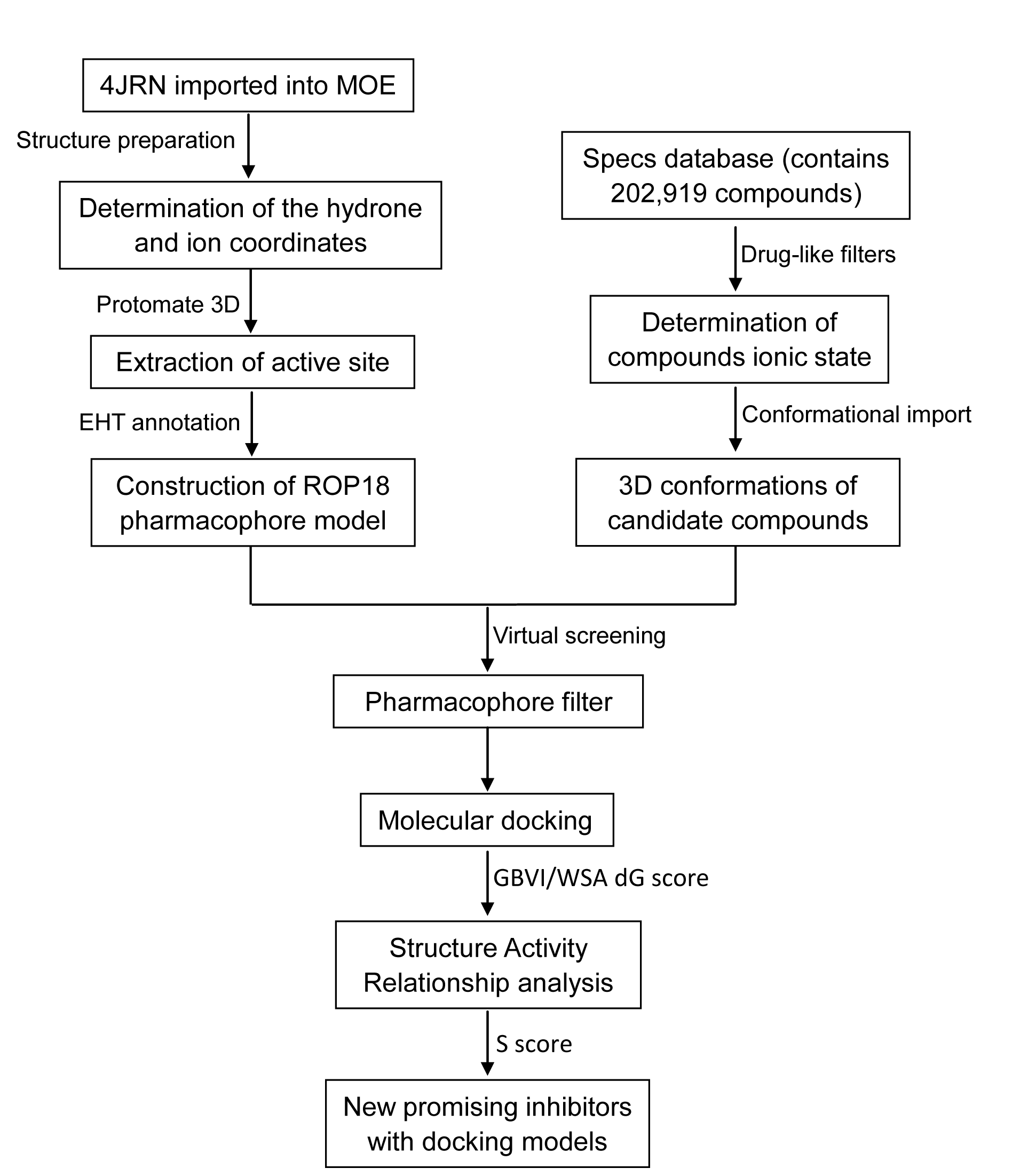

Supplement: Supplementary file 3 — Additional file 3: Figure S3. Virtual screening protocol of the structure based approach for ROP18 pharmacophore model construction and competitive inhibitors identification. [file 13071_2019_3341_MOESM3_ESM.tiff]

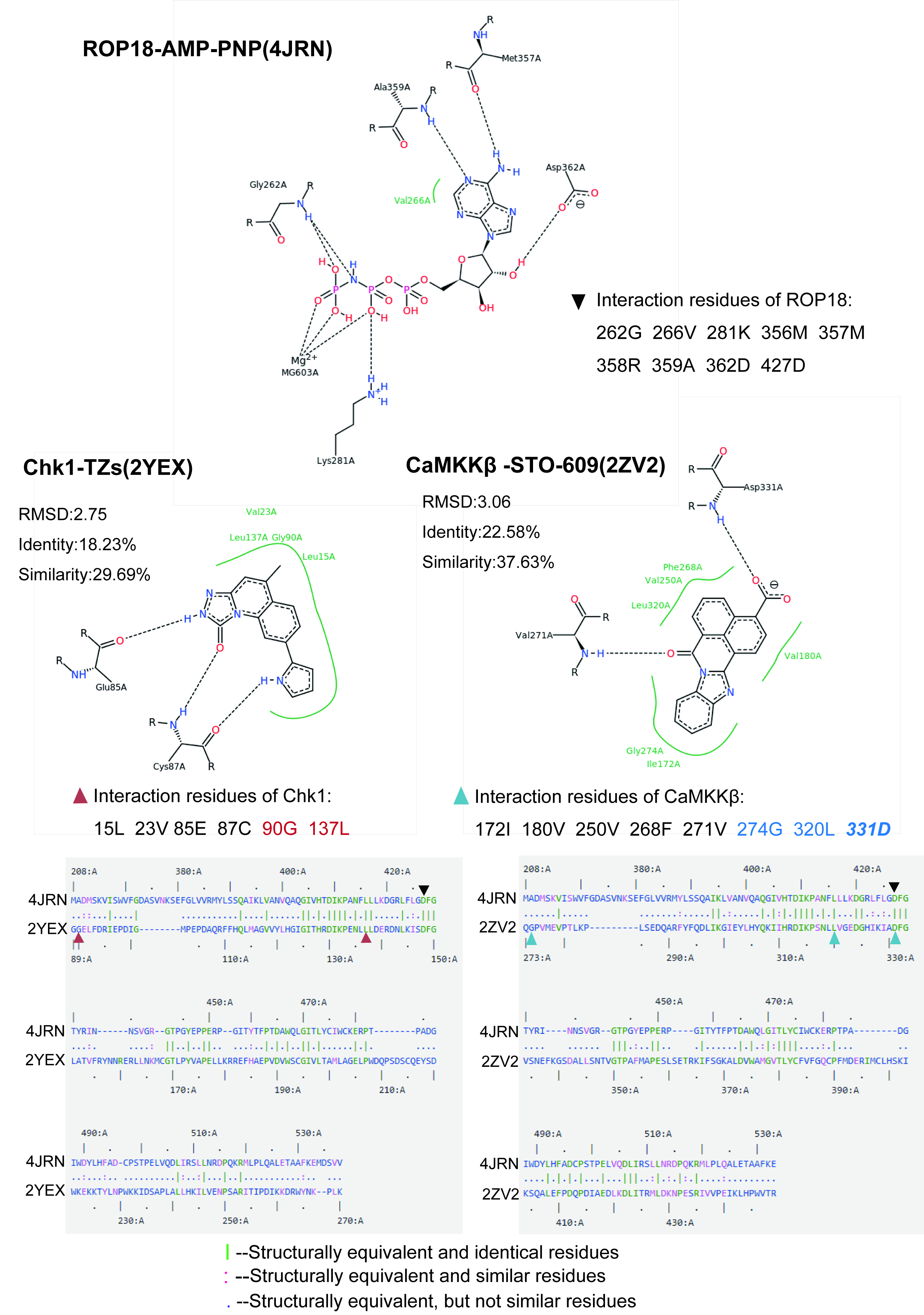

Supplement: Supplementary file 4 — Additional file 4: Figure S4. Interaction schematic diagrams of three structures of kinase-ligand complexes (4JRN, 2YEX and 2ZV2), the structure similarity and sequence alignment of the KDs of human kinases Chk1/CaMKKβ and ROP18, respectively. The black dashed lines in the interaction schematic diagrams indicate hydrogen bonds, salt bridges and metal interactions. Green solid lines show the hydrophobic interactions and green dashed lines show π-π and π-cation interactions. Abbreviations: KD, kinase domain; Chk2; checkpoint kinase 2; CaMKKβ, calcium/calmodulin dependent protein kinase kinase 2 beta; RMSD, root-mean-square deviation. [file 13071_2019_3341_MOESM4_ESM.tiff]
